# Chloroform

**Published:** 1848-02

**Authors:** 


### CHLOROFORM.

This new anæsthetic agent seems nearly or quite to have superseded ether in the minds of the profession, in London and Edinburgh particularly. Thus far, reports of its utility are discordant, but the general sentiment appears to be favourable to its employment in preference to ether. In Philadelphia it has likewise been employed to some extent, and with a like want of uniformity in results. Recently, at the clinic of the Jefferson Medical College, Professor Pancoast removed a large tumour with a considerable portion of the upper maxillary bone, while the patient was under the influence of the chloroform. The patient struggled a good deal during the operation, but declared she had not suffered pain from its performance. We are informed by Professor Gilbert, of the Pennsylvania Medical College, that he has employed it in ten instances with success. The operations were for the extraction of teeth, removal of various tumours, and the amputation of a cancerous breast. Neither vomiting nor convulsions ensued. In one case, the patient, who was of a very nervous temperament, and at the time threatened with symptoms of hysteria, directly after inhaling the chloroform, fell into a paroxysm of that affection, from which, however, she recovered without anything untoward.

Since writing the above, we have seen a beautiful pamphlet of 100 pages, entitled "Etherization, with Surgical Remarks," by Professor John C. Warren, M. D., of Boston, which we recommend to the sceptical on this subject. The etherizations, on which his remarks are founded, amounted to at least *two hundred*, and were practised in the presence of "gentlemen qualified to judge of the accuracy of the particular and general statements." The general conclusions of Dr. Warren are as follows:

"*First.* Inhalation of ether produces insensibility to pain.

"*Secondly.* Ethereal insensibility, judiciously effected, is not followed by any dangerous consequences.

"*Thirdly.* Its administration is easy, and usually requires but a few minutes.

"*Fourthly.* Individuals of all ages may be safely etherized.

"*Fifthly.* Individuals of the same age are susceptible of the influence of ether in variable degrees.

"*Sixthly.* Surgical operations may be done under the effect of ether, which could not be done without.

"*Seventhly.* Operations very short, and not very painful, especially those about the head and neck, are best done without ether.

"*Eighthly.* The shock of the nervous system is greatly diminished by etherization.

"*Ninthly.* The use of ether has increased the number of successful operations, by encouraging a resort to them at an earlier period of the disease.

"*Tenthly.* The use of the sponge is more safe and easy than that of any special apparatus.

"*Eleventhly.* A special apparatus is convenient for some peculiar cases.

"*Twelfthly.* The existence of chronic pulmonary disease rarely forms an objection to etherization.

"*Thirteenthly.* Etherization may often be employed advantageously as a substitute for narcotics.

"*Fourteenthly.* The employment of ether does not retard the healing of wounds, nor give them an unfavourable character.

"*Fifteenthly.* The pains of death may often be relieved by etherization."

Under all the evidence, then, which has been brought forward in favour of the employment of these anæsthetic agents in surgical operations, it becomes a momentous question—on which we have no hesitation in recording our sentiments in the negative—whether the philanthropic surgeon can in any respect be justified in refusing to employ them in cases in which the experience of observers, and many of them the most enlightened and trustworthy of the profession, have had recourse to them with undoubted safety and as undoubted benefit.

